# Microglia dysfunction drives disrupted hippocampal amplitude of low frequency after acute kidney injury

**DOI:** 10.1111/cns.14363

**Published:** 2023-07-19

**Authors:** Ziyang Yu, Huize Pang, Yifan Yang, Doudou Luo, Haiping Zheng, Zicheng Huang, Mingxia Zhang, Ke Ren

**Affiliations:** ^1^ School of Medicine Xiamen University Xiamen China; ^2^ Department of Radiology The First Hospital of China Medical University Shenyang China; ^3^ State Key Laboratory of Cellular Stress Biology, Innovation Center for Cell Signaling Network, School of Life Sciences Xiamen University Xiamen China; ^4^ State Key Laboratory of Molecular Vaccinology and Molecular Diagnostics and Center for Molecular Imaging and Translational Medicine, School of Public Health Xiamen University Xiamen China

**Keywords:** acute kidney injury, apoptosis, microglia, neuron, Rs‐fMRI

## Abstract

**Aims:**

Acute kidney injury (AKI) has been associated with a variety of neurological problems, while the neurobiological mechanism remains unclear. In the present study, we utilized resting‐state functional magnetic resonance imaging (rs‐fMRI) to detect brain injury at an early stage and investigated the impact of microglia on the neuropathological mechanism of AKI.

**Methods:**

Rs‐fMRI data were collected from AKI rats and the control group with a 9.4‐Tesla scanner at 24, 48, and 72 h post administration of contrast medium or saline. The amplitude of low‐frequency fluctuations (ALFF) was then compared across the groups at each time course. Additionally, flow cytometry and SMART‐seq2 were employed to evaluate microglia. Furthermore, pathological staining and Western blot were used to analyze the samples.

**Results:**

MRI results revealed that AKI led to a decreased ALFF in the hippocampus, particularly in the 48 h and 72 h groups. Additionally, western blot suggested that AKI‐induced the neuronal apoptosis at 48 h and 72 h. Flow cytometry and confocal microscopy images demonstrated that AKI activated the aggregation of microglia into neurons at 24 h, with a strong upregulation of M1 polarization at 48 h and peaking at 72 h, accompanying with the release of proinflammatory cytokines. The ALFF value was strongly correlated with the proportion of microglia (|*r*| > 0.80, *p* < 0.001).

**Conclusions:**

Our study demonstrated that microglia aggregation and inflammatory factor upregulation are significant mechanisms of AKI‐induced neuronal apoptosis. We used fMRI to detect the alterations in hippocampal function, which may provide a noninvasive method for the early detection of brain injury after AKI.

## Graphical abstract

Flow schematic of the experiment design. The whole study consisted of two groups at baseline, 24 h, 48 h, and 72 h. The baseline point is prior to CM or saline tail injection. After performing Morris water maze test, then the fMRI data were collected.

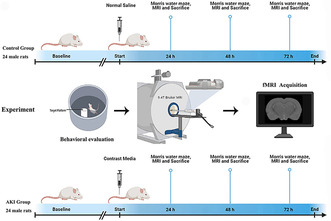

## 1 INTRODUCTION

Acute kidney injury (AKI) is a common condition that is characterized by a sudden decrease in renal function within 48 h.^1^, ^2^ Studies have demonstrated that AKI can have a detrimental effect on the central nervous system, leading to symptoms such as irritability, attention deficits, altered mental status, seizures, and even death.^3^, ^4^ Compared to chronic kidney disease (CKD), AKI patients are more vulnerable to encephalopathy due to the lack of time for adaptation.^5^, ^6^ Dementia is one of the most serious neurological outcomes of AKI, and a 12‐year follow‐up study in a larger population has revealed that AKI significantly increases the risk of dementia.^7^ Mild cognitive impairment (MCI) is a transitional state between normalcy and dementia, and it is therefore essential to identify and address MCI in AKI patients in order to reduce the incidence of adverse brain events.^8^, ^9^ Unfortunately, many AKI patients with MCI are often overlooked due to negative conventional neuroimaging.^10^, ^11^, ^12^ As the population of AKI patients continues to grow, it is important to bring attention to this clinical phenomenon in order to reduce the incidence of neurological complications.

Neuroimaging research has significantly enhanced our comprehension of the neurological modifications connected to AKI. For instance, previous studies have found that periventricular and white matter hyperintensities in MRI are linked to neuronal loss, demyelination, and gliosis.^13^, ^14^ Despite this, the neuropathological mechanisms of cognitive impairment after AKI remain largely unknown. Resting‐state functional MRI (rs‐fMRI) has been proposed as a viable tool for evaluating brain abnormalities in AKI, as it is a blood oxygen saturation‐dependent contrast that can detect brain functional alterations in an early stage.^15^ Among the various rs‐fMRI methods, the amplitude of low‐frequency fluctuations (ALFF) has been proven to be an efficient and reliable index for uncovering spontaneous neuronal early metabolic activity.^16^, ^17^ As the ALFF in fMRI is thought to reflect the intensity of spontaneous activity during the resting state, it has been utilized to evaluate regional neuronal activity in other cognitive dysfunction diseases.^18^, ^19^ However, only a few studies have used it in AKI. Therefore, we applied rs‐fMRI to detect the brain functional alterations in AKI.

Studies have demonstrated that AKI can lead to changes in inflammatory cytokines in the cerebral cortex and hippocampus, which are associated with cognition.^20^ Salama et al. found that AKI was associated with an increased expression of TLR4 in the hippocampus and striatum.^21^ Although these studies provide insight into the cognitive effects of kidney damage, the mechanisms behind neurological changes following AKI are still not fully understood. It has been observed that inflammation in the central nervous system (CNS) can lead to neuronal cell death and cognitive impairment in neurological diseases.^22^, ^23^ Microglia, as the first immune cells to be activated in an inflammatory response, can contribute to neuron death by releasing cytokines under inflammatory conditions.^24^, ^25^ Evidence has mounted to suggest that the occurrence of acute‐stage cell death leads to an increased proportion of microglial cells to neurons, supporting that microglial dysfunction is a contributing factor in the pathogenesis of prevalent brain disorders.^26^ Recent studies have demonstrated that neuroinflammation is a crucial risk factor that has a direct impact on the emergence of cognitive impairment.^27^, ^28^ Furthermore, microglial activation is a key contributor to neuronal loss in neurodegenerative conditions.^29^ Therefore, targeting microglia may be a potential strategy to reduce neuroinflammation and a promising strategy to treat the cognitive dysfunction.^30^ However, the role of microglia in AKI‐brain disease is still unclear, and effective biomarkers to quantify microglia are lacking. Quantifying microglia is essential for early targeted anti‐inflammatory treatments to protect neurons in clinical AKI, with a particular focus on rs‐fMRI.

The purpose of this research was to analyze the changes in rs‐fMRI in AKI, with a focus on the clinical applicability of rs‐fMRI. Moreover, the ALFF index was employed to measure the microglial density and to identify the mechanism of the AKI‐brain axis. We hypothesized that AKI patients may have distinct functional activities, which are likely to be associated with cognitive impairment.

## 2 MATERIALS AND METHODS

### 2.1 Animals and treatment

**FIGURE 1:**
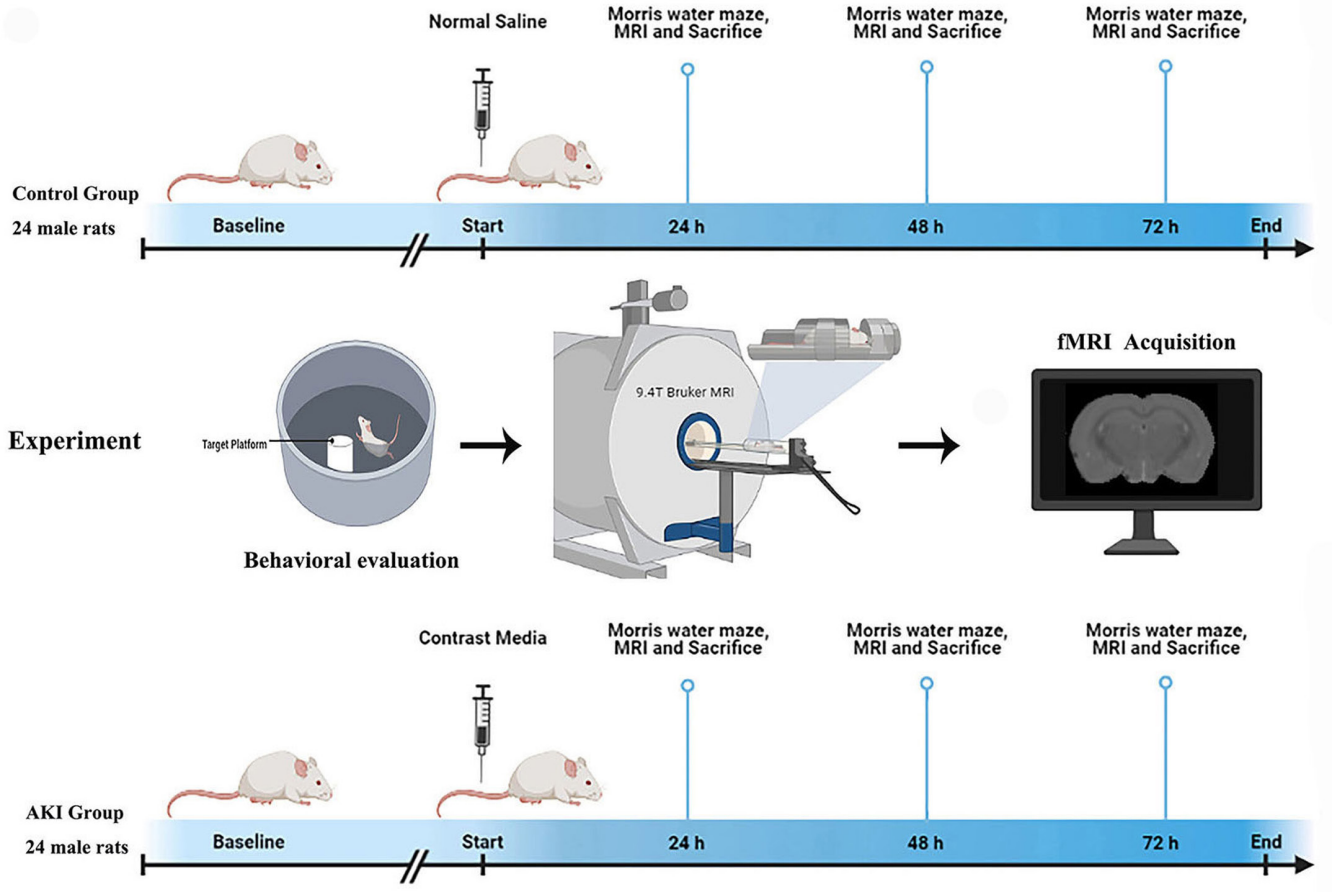
Flow schematic of the experiment design. The whole study consisted of two groups at baseline, 24 h, 48 h, and 72 h. Baseline point is prior to CM or saline tail injection. After performing Morris water maze test, the fMRI data were collected.

Forty‐eight adult male Sprague–Dawley rats (age 6–8 weeks; weight 200–250 g) were obtained from the Laboratory Animal Center of Xiamen University and housed in a controlled environment with a temperature of 22 ± 2°C, relative humidity of 50%–70%, and a 12‐h light/dark cycle, with free access to food and water. All studies were approved by the Animal Care and Ethics Committee of Xiamen University. The rats were randomly divided into two primary groups: a sham control group with saline and an acute kidney injury (AKI) model group. The AKI model was induced by an iodinated contrast agent as described in a previous study. The two groups were further subdivided into four subgroups at baseline, 24, 48, and 72 h, with six rats per subgroup. A schematic of the study groups and regimens is presented in Figure 1.

### 2.2 Magnetic resonance imaging acquisition

All MRI was acquired on a horizontal bore 9.4‐Tesla Bruker BioSpec system with a BGA‐9S gradient insert. Images were acquired using a standard Bruker cross coil setup with a quadrature volume coil and a quadrature hear coil for rats. The rats were anesthetized (2.0% isoflurane mixed with medical air) and mechanically ventilated. The MR‐compatible small animal monitoring and heating system were used to keep the rectal temperature, arterial blood pressure, and breath rate within normal ranges. End‐tidal carbon dioxide values were adjusted to the level of 30–40 mmHg.^31^ Three orthogonal multi‐slice Turbo RARE T2‐weighted images were acquired for each rat to guarantee brain‐positioning uniform (repetition time 2000 ms, echo time 15 ms, 15 slices of 0.5 mm) and field shimming was used to render field homogeneity. For anatomical referencing, a T2‐weighted RARE pilot image was taken in the midsagittal plane to localize the anterior commissure. T2‐weighted anatomical images were obtained by using a RARE sequence^32^ (scan parameters: TR = 5500 ms, TEeff = 33 ms, slice thickness = 0.5 mm, matrix size = 256 × 256, FOV = 40 × 40 mm2, NEX = 4). Resting‐state data were acquired using a gradient‐echo EPI sequence,^33^ (scan parameters: TR = 2000 ms, TE = 20 ms, 200 volumes and a total rs‐fMRI acquisition time of over 6.5 min, bandwidth = 350 kHz, flip angle = 30°, slice thickness = 0.70 mm without slice gap, matrix size = 96 × 96, and FOV = 4.00 × 4.00 cm^2^).

### 2.3 MRI data pipeline

SPM12 was performed for fMRI data preprocessing on MATLAB. The main steps were as follows: (I) removal of the first five volumes; (II) slice timing and realigned to the first image by a least‐squares approach and a six‐parameter (rigid body) spatial transformation; (III) all fMRI data were normalized to standard template (EPI template for rats). The normalization steps were performed on the ANTs (advanced normalization tools), which consist of a global 12‐parameter affine transformation followed by the estimation of the nonlinear deformations; (IV) nuisance covariates regression (the Friston‐24 parameters, signals from white matter, and cerebrospinal fluid); (V) linear detrend; (VI) spatial smoothing using a Gaussian kernel full width at half maximum (FWHM = 0.6 mm for rats). After preprocessing steps, voxel‐wise mean ALFF maps of rats were generated using DPABI software.

### 2.4 Morris Water Maze

Cognitive capacity and spatial memory were tested using the Morris water maze procedure.^34^ The water maze is 120 cm in diameter, 35 cm in height and has a circular target platform 10 cm in diameter. The water maze is divided into four quadrants (including north, south, east, and west) and the target platform is fixed in the western quadrant with the platform height 2 cm below the water surface. The water temperature was maintained at 25°C by the air conditioner and the water was blackened with ink. The humidity, light, and sound were properly maintained as the protocol of previous studies. A camera was placed overhead the water pool and was attached to the Noldus XT14 software for assessment.

As described in previous research,^35^ during the training phase, the rats were placed in the pool from all four quadrants and given 60 s to find the target platform. If the rats could not find the target platform within 60 s, we used a rod to guide them to the platform. After reaching the target platform, rats were allowed to stay there for 10 s to solidify their learning and memory, and then they were removed from the maze. The time taken from the starting point to the target platform and the track were recorded. This method was applied for seven consecutive days of training, and the examination was performed on the eighth day. Then, the AKI model was built and tested at 24 h, 48 h, and 72 h, respectively. In the test phase, the platform was removed and the rats were placed in the pool from the southern quadrant (opposite to target zone) for 1 min. The trace and the stay ratio of the target quadrant were recorded.

### 2.5 Sample collection

Following MRI and Morris Water Maze, the rats were euthanized with an overdose of anesthetics. Blood from the femoral artery was collected into tubes with separation gel and then was centrifuged at 4°C, 4000 rpm for 10 min. The left kidneys were removed at the renal hilum for histopathological analysis. The brains were collected for enzyme‐linked immunosorbent assay (ELISA) analysis, western blot, flow cytometry, and immunofluorescence analysis.

### 2.6 Hematoxylin and eosin staining

After the kidneys were fixed in 4% paraformaldehyde at room temperature (RT) for 24 h, they were dehydrated with an increasing gradient of ethanol and dimethylbenzene and then embedded in paraffin. Paraffin kidney sections with 4‐mm thickness were stained with the kit according to the manufacturer's instructions (H&E Staining Kit, C0105S, Beyotime, China). The kidney specimens were evaluated according to the presence of tubular desquamation, dilatation, vacuolation, necrosis, and interstitial infiltration.

A total of three fields of each slice were randomly selected for a blinded examination under a microscope (×20 magnification). The evaluation of the specimens was based on the ATN scoring principle, graded by proximal tubule dilation, brush border damage, proteinaceous casts, interstitial widening, and necrosis (total score range from 0 to 5; 0, none; 1, <11%; 2, 11%–25%; 3, 26%–45%; 4, 46%–75%; 5, >75%).^36^

### 2.7 Immunohistochemistry

The paraffin sections after deparaffinization were performed in 100°C citrate antigen retrieval solution for 30 min for antigen retrieval and cooled to RT naturally. 3% H_2_O_2_ in PBS (Phosphate Buffer solution) was used to block the samples’ endogenous peroxidase activity, washed three times in PBS, and then pretreated with Triton‐X 100 for 30 min at RT. After washed in PBS, the samples were blocked in 10% normal goat serum in PBS for 1 h in RT. The sections were immunolabeled by incubating with KIM‐1 antibodies at 4°C overnight specific antibodies. Sections were then washed with PBST for three times. Thereafter, they were incubated with the horseradish peroxidase (HRP)‐labeled specific secondary antibodies for 30 min. Sections development was done in a 3,3′‐diaminobenzidine (DAB) kit. We randomly obtained three photomicrographs per section from each rat for the final quantitative analysis of each group.

### 2.8 Measurement of proinflammatory cytokines by ELISA

According to the MRI results, the hippocampus samples were sonicated (10 w, 2 × 5 s) in PBS buffer (20 mg/3 mL) and then centrifuged at 12,000r for 10 min at 4°C. Interleukin‐1β and Interleukin‐6 were measured in the supernatant by ELISA (R&D Systems, United States) according to the instructions of manufacturers.

### 2.9 Western blot

Total protein was isolated from different brain regions in the tissue protein extraction reagent. The protein concentration of each sample was measured by the bicinchoninic acid (BCA) method. A molecular weight marker (5 μg/lane) and the protein samples (30 μg/lane). Equal amounts of protein were separated on a suitable sodium dodecyl sulfate (SDS)‐polyacrylamide gel and transferred to a polyvinylidene difluoride (PVDF) membrane by wet transfer, which was immediately blocked with 5% skim milk for 1 h at RT. Then, the membrane was incubated overnight at 4°C with corresponding primary antibodies at 4°C overnight. Antibodies reactive with NeuN (1:1000), Bax (1:1000), Bcl‐2 (1:1000), caspase3 (1:1000), β‐actin (1:1000), and GADPH (1:1000). The antibody information is listed in Table S1. After being washed with TBST buffer, the membrane was incubated with HRP‐linked secondary antibodies at RT for 60 min. The protein bands were visualized with an enhanced chemiluminescence (ECL) kit by a chemiluminescence system. Beta‐action was the internal control. The density of protein bands was analyzed with ImageJ software (NIH, Bethesda, MA, USA).

### 2.10 Immunofluorescence

The animals (three rats per group) were anesthetized and transcardial perfusion with PBS. Then the whole brain was submitted to 4% PFA for fixation and dehydrated with an increasing gradient of sucrose. Fixed tissues embedded in OCT and sectioned into a thickness of 25 μm coronal slices with a Vibratome (VT1000S; Leica).

Following pretreating with Triton‐X 100 and blocking with 10% normal goat serum in PBS, the brain sections were incubated with primary antibodies for corresponding primary antibodies at 4°C overnight Iba‐1, NeuN, Bax, Bcl‐2, and caspase3. After washing three times with PBST, the brain sections were incubated with appropriate secondary antibodies. Next, these brain slices were covered with anti‐fading mounting medium containing 4,6‐diamino‐2‐phenylindole (DAPI, Southern Biotech). Finally, the brain sections were observed under a fluorescence microscope (Olympus BX50/BX‐FLA/DP70, Olympus Co., Tokyo, Japan).

### 2.11 Flow cytometry

The rats (three rats per group) were anesthetized and transcardial perfusion with PBS. Different brain regions were separated into 70 μm strainer, homogenized by the needle bladder, and collected into tubes. Deoxyribonuclease (D4527‐10KU, Sigma, 50,000 kU/mL) was added to the tubes and then centrifuged at 4°C, 1500 rpm for 10 min. The sediment was resuspended with 30% Percoll. The suspension was poured slowly down the inner wall of the tubes with 70% Percoll so that the different density of Percoll clearly separated. The cocktail was centrifuged gradient 30 min at 600 G (20°C) with no brake. Then collected 2.0 ± 0.5 mL of the interphase between 30% and 70% Percoll into a clean 15 mL conical tube and added isopycnic HBSS. The samples were centrifuged at 4°C 2000 rpm for 5 min and the sediment was the target leukocytes. The cells were then incubated with CD marker antibodies (CD45, 1:200, BB700, OX‐1, BD Pharmingen, United States; CD11b, 1:200, FITC, WT.5, BD Pharmingen, United States; CD86, 1:200, BV650, 24F, BD Pharmingen, United States) for 30 min at 4°C in darkness and were analyzed by flow cytometry (Bechman, MoFlo Astrios EQS).

### 2.12 3' mRNA‐Sequencing Library Preparation

The microglia (CD11b^high^ and CD45^int^) were collected from the flow cytometry and washed twice by 1xDPBS through centrifugation at 4°C, 1500 rpm for 5 min. Add cell lysate (final concentration: 160 mM Tris pH 7.5, 0.16% Sarkosyl, 16 mM EDTA, 0.5 U/μL RNase Inhibitor) to fully lyse the collected cells and then add reverse transcription mix (1x RT buffer, 1 mM dNTPs, 1 U/μL RNase Inhibitor, 2.5 μM Template_Switch_Oligo, and 10 U/μL, Maxima H‐RT). Incubated the mix at 42°C for 90 min to generating cDNA. The cDNA was then amplified as follows: 95°C for 3 min; then 24 cycles of 98°C for 20 s, 58°C for 20 s, and 72°C for 3 min. The PCR was performed in 50 μL reaction volumes with 1x PCR Buffer, dNTP mix, PCR primer, DNA polymerase, and nuclease‐free water. The PCR products are purified using 0.6x DNA Clean Beads and quantified by qubit3.0. The 3′‐end enriched sequencing library was prepared using Tn5 transposase and amplified as follows: 72°C for 3 min, 98°C for 30 s; and 13 cycles of: 98°C for 15 s, 55°C for 30 s, and 72°C for 30 s. The 3′‐end enriched library products are purified using 0.6x DNA Clean Beads and quantified by qubit3.0. The fragment size of the 3′‐end enriched sequencing library is analyzed by Agilent‐4150, and the average size is between 450 and 650 bp. The libraries are sequenced on the Illumina NovaSeq 6000 according to the manufacturer's instructions. Read 1 is 150 bp and read 2 is 150 bp for all the experiments.

### 2.13 Statistical analysis

The Kolmogorov–Smirnov test was used to assess the normality of the datasets in each group. Parametric data were expressed as the mean ± SEM and analyzed using a one‐way ANOVA test, followed by Bonferroni post hoc tests to compare the baseline values with different time points within a group or a two‐tailed t test for single comparison between two groups at the same time. The Mann–Whitney *U* test was used for the nonparametric data, and Spearman correlation analysis was employed to investigate the relationship between fMRI variables and the microglia percentage. All statistical analyses were conducted using SPSS 22.0 (SPSS Inc. IBM), with a two‐tailed *p* value of <0.05 considered significant.

## 3 RESULTS

### 3.1 Rs‐fMRI analysis for rats

#### 3.1.1 Effects of AKI on BOLD response and ALFF of brain

**FIGURE 2:**
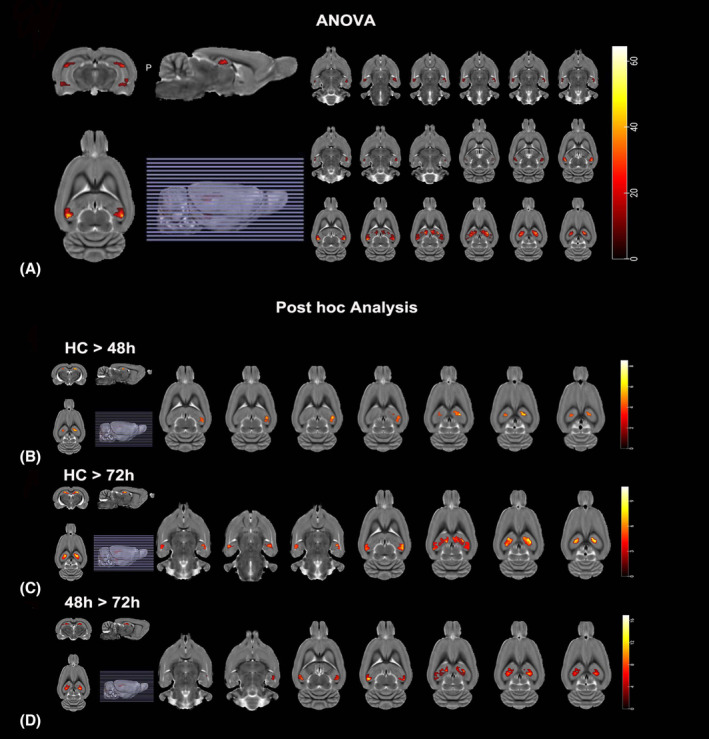
The effect of AKI on the brain maps of rs‐fMRI. The left side of the image corresponds to the left side of the brain in axial orientation; slice coordinates according to SIGMA rat brain templates are shown in the upper left corner of the slices, indicating Z‐axis in axial orientation. (A) Brain maps for amplitude of low‐frequency fluctuations (ALFF) differences among different groups. The significant level was set at *p* < 0.05 and cluster size >47 voxels within the group mean gray mask, which corresponded to a corrected *p* < 0.05. (B) Brain maps for ALFF difference between the AKI at 48 h and HC group. Red and yellow color stand for increased ALFF. The significant level was set at *p* < 0.05 and cluster size >19 voxels, which corresponded to a corrected *p* < 0.05. (C) Brain maps for ALFF difference between the AKI at 72 h and HC group. Red and yellow color stands for increased ALFF. The significant level was set at *p* < 0.05 and cluster size>41 voxels, which corresponded to a corrected *p* < 0.05. (D) Brain maps for ALFF difference between the AKI at 48 h and 72 h group. Red and yellow color stands for increased ALFF. The significant level was set at *p* < 0.05 and cluster size >14 voxels, which corresponded to a corrected *p* < 0.05.

The AKI group exhibited ALFF differences in five clusters located in the left CA1, left DG, and right CA1, as illustrated in Figure 2A. The mALFF value of the cluster at different time points is outlined in Table 1.

**TABLE 1:** Brain regions showing significant ALFF differences among the groups and after post hoc.

Brain regions	Cluster	Peak SIGMA coordinate			*T* Value
		*X*	*y*	*z*	
ANOVA results					
CA1_R	47	61	−59.05	6.2	24.2557
CA1_L	63	−59	−62.05	6.2	30.3341
CA1_R	153	52	−65.05	36.2	69.6254
CA1_L	75	−53	−62.05	36.2	62.1723
Post hoc results					
HC > 48 h					
CA1_L	36	−44	−47.05	39.2	7.2525
CA1_R	19	25	−41.05	45.2	6.2532
CA1_L	37	−32	−35.05	42.2	11.6192
HC > 72 h					
CA1_R	41	64	−50.05	−2.8	8.3107
CA1_L	58	−56	−50.05	0.20	8.9508
CA1_R	134	16	−38.05	39.2	13.3225
CA1_L	74	−56	−59.05	33.2	9.9909
DG_L	90	−20	−29.05	45.2	11.3420
48 h > 72 h					
CA1_L	14	−59	−59.05	6.2	7.7778
CA1_R	119	49	−59.05	33.2	20.2759
CA1_L	30	−53	−62.05	33.2	12.3424
DG_L	54	−32	−35.05	39.2	17.2306

*Note*: A positive T value represents decreased ALFF value in AKI rats.

Abbreviations: CA1, cornu_Ammonis_1; DG, Dentate Gyrus; L, R, Left and Right.

#### 3.1.2 Post hoc ALFF comparisons

Analysis of the control group versus the rats with acute kidney injury (AKI) revealed no statistically significant differences in the amplitude of low‐frequency fluctuations (ALFF) at 24 h. This result was consistent with the behavioral tests. At 48 h, the AKI rats had three clusters of significantly lower ALFF values in the left CA1, left DG, and right CA1 regions of the brain, with the largest cluster being in the right CA1, containing 36 voxels (Table 1, Figure 2B). At 72 h, five clusters of significantly lower ALFF values were detected, with the largest being in the left CA1, containing 119 voxels (Table 1, Figure 2C). Additionally, the right primary visual cortex, right CA1, and left DG regions had lower ALFF values at 72 h compared to 48 h (Table 1, Figure 2D). Overall, the behavioral tests and MRI results suggest that AKI progressively worsens and expands the extent of brain injury.

### 3.2 AKI rat assessment

#### 3.2.1 Renal function and cytokines

**FIGURE 3:**
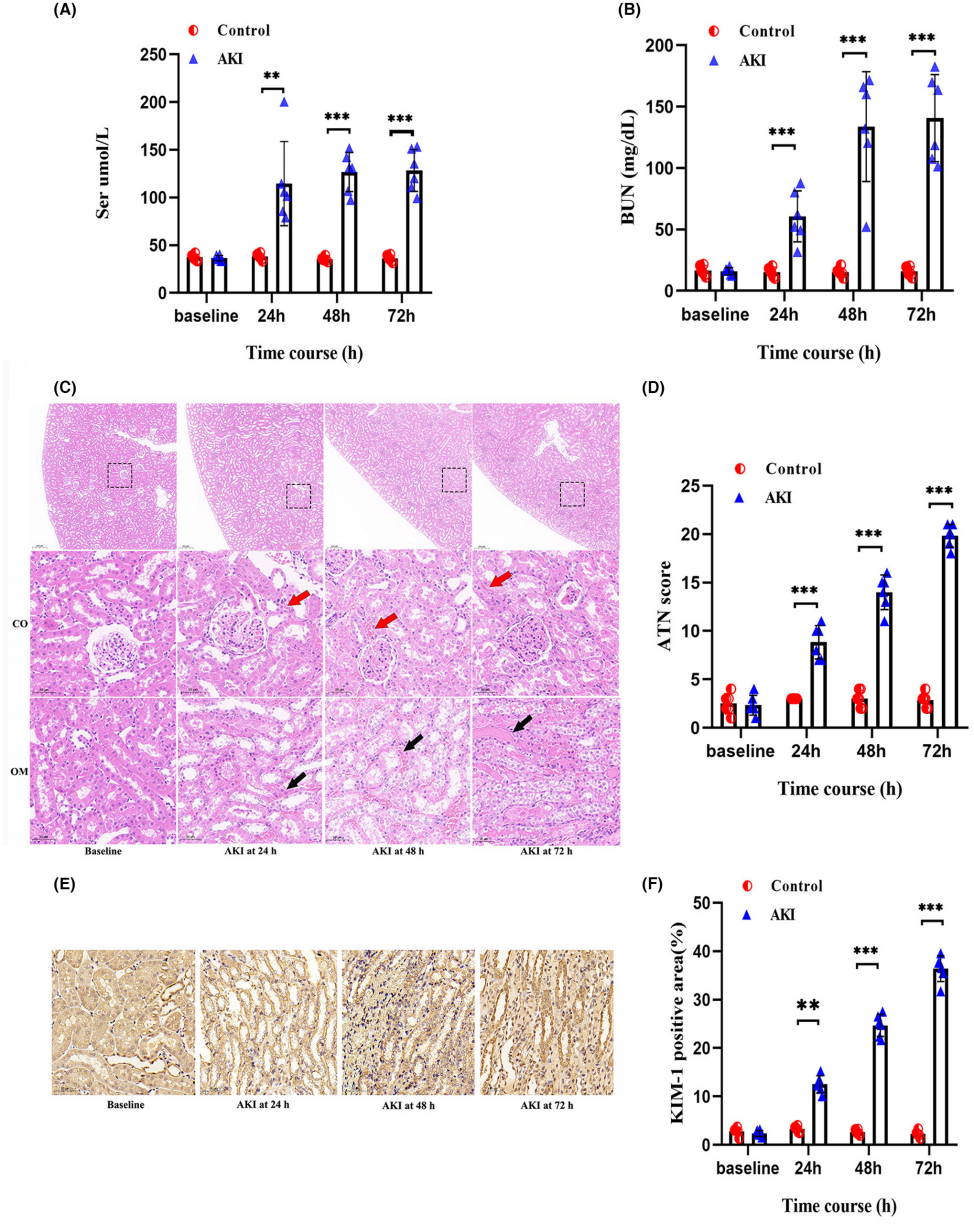
Evaluation of the rat AKI models. The serum creatinine (A) and blood urea nitrogen (B) were measured via tail vein at each point to evaluate the modeling. (C) Hematoxylin–eosin‐stains of the corticomedullary junction area showed the representative kidney injury process in the AKI group. Red arrowheads indicate brush border; black arrowheads indicate intraluminal debris. CO, cortex; OM, outer medulla; Scale bars, 50 μm. (D) ATN scores in the kidneys of control group (white bars) and AKI group (black bars) rats on the indicated day (*n* = 6 per group). (E) The representative photomicrographs showing immunohistochemical analysis for KIM‐1 in the kidneys of AKI rats on the indicated day. (*n* = 6 per group). KIM‐1‐*positive cells were mainly* occurred in the OM and the signals were visualized by horseradish peroxidase (HRP) and a substrate chromogen (3,3′‐diaminobenzidine; DAB). Scale bars, 50 μm. (F) Quantification of KIM‐1 expression in the kidneys of control group (white bars) and AKI group (black bars) rats on the indicated day (*n* = 6 per group). All data in this figure are analyzed using parametric test. Data are presented as means ± SD. *p < 0.05, **p < 0.01, ***p < 0.001.

A significant increase in serum creatinine and blood urea nitrogen was detected in AKI rats from 24 h when compared with the control group (Figure 3A,B).

#### 3.2.2 Renal Histopathology Analysis

The changes in CO and OM on H&E in the AKI rats revealed widespread tubular degeneration and severe necrosis of renal tubules, tubular vacuolation, and dilation (Figure 3C). As compared to the control group, in the first 24 h after CM injection, the nuclei started to fragment and the proximal tubule dilated. Further, at 48 and 72 h, the proteinaceous casts became larger and the interstitial infiltrated. Notably, the histological injury scores differed significantly in the AKI group compared to the control group (Figure 3D).

#### 3.2.3 KIM‐1 Immunohistochemistry Results

KIM‐1 (kidney injury molecule‐1), a phosphatidylserine receptor, primarily mediates the phagocytosis of apoptotic bodies and oxidized lipids by tubular cells.^37^ Compared with the control group, KIM‐1 increased continuously from 24 h to 72 h (Figure 3E) and its quantitative analysis is presented in Figure 3F. The results of ATN scores and KIM‐1 expression revealed that the most severe tubular cell damage occurred at 72 h.

### 3.3 Deficits in cognitive function induced by AKI rats

**FIGURE 4:**
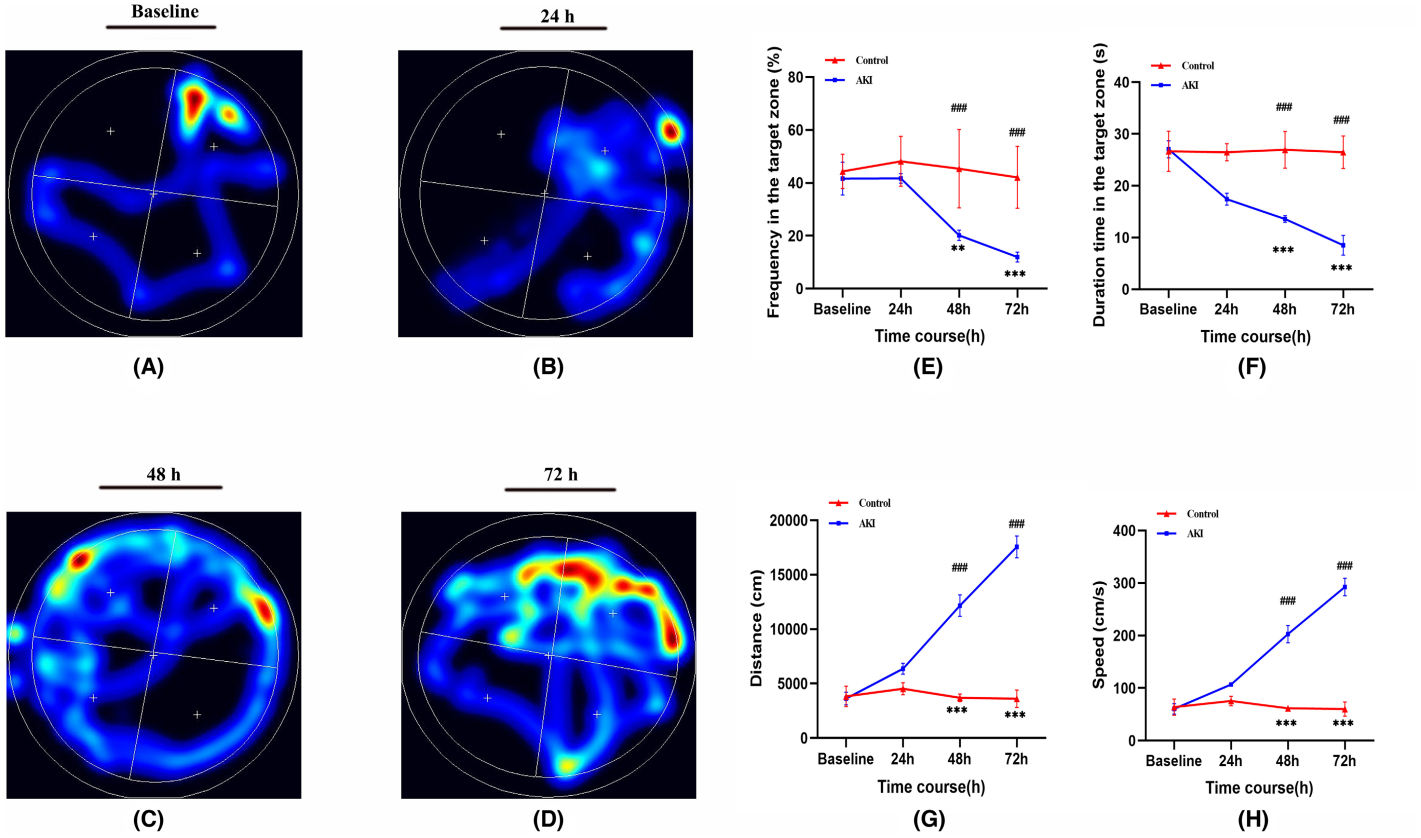
Effect of AKI on the ability of spatial learning and memory in AKI was detected by the Morris water maze. (A–D) Represent the swimming trajectories of rats in each group on the indicated day. (E) Proportion of time spent on the target zone in each group (*n* = 6 per group). (F) Duration time spent on the target zone in each group. (G) Distance of swimming trace in each group. (H) Swimming speed in each group. Data are presented as means ± SD. *p < 0.05, **p < 0.01, ***p < 0.001.

We conducted a water maze test to investigate whether AKI induced spatial learning and memory impairment. The test was evaluated using the hidden platform and the residence ratio of the target quadrant. The results showed that there was no significant difference between the control group and AKI rats at 24 h. However, the residence ratios of the AKI rats were significantly decreased at 48 h and 72 h, ranging from 45.36% to 20.19% (48 h) and 42.12% to 11.95% (72 h). Additionally, the duration time spent on the target zone was significantly reduced at 48 h and 72 h, ranging from 26.93 s to 13.59 s (48 h) and 26.48 s to 8.5 s (72 h). Moreover, swimming distance and speed were significantly increased in AKI rats at 48 h and 72 h. These findings demonstrate that AKI can cause spatial learning and memory impairment in rats (Figure 4).

### 3.4 AKI induced microglia activation in hippocampus of rats

**FIGURE 5:**
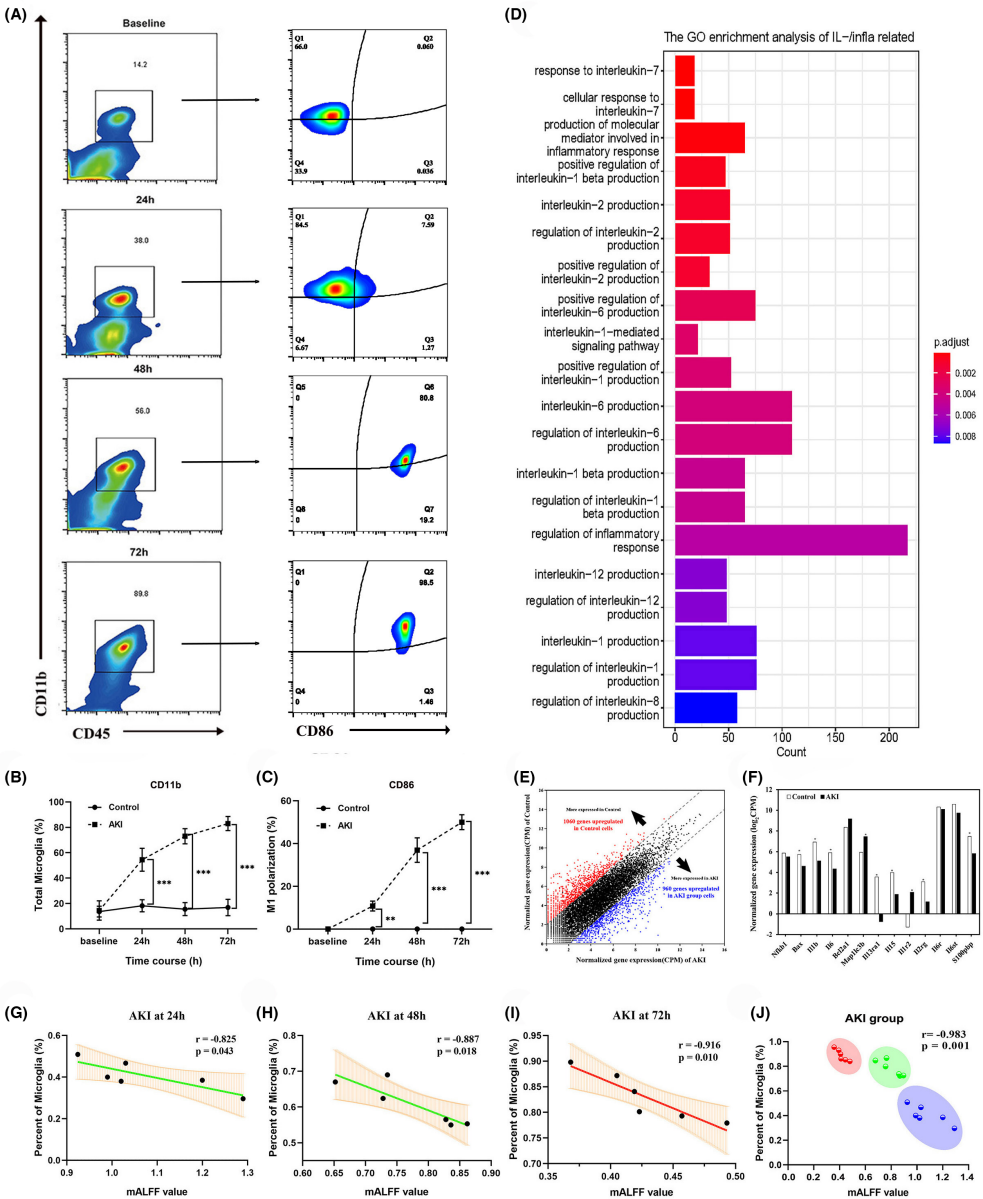
The effect of AKI on microglial activity. (A) The representative photomicrographs showing activated microglia from the hippocampus of the control and AKI groups. Flow cytometry analysis of the frequency of microglia cell (CD11b^+^CD45^int^) and the M1 polarization (CD86^+^). (B) Gene ontology (GO) enrichment analysis of differentially expressed genes (DEGs) common to the control and AKI group comparisons. (C, D) Quantification of subsets in microglia and M1 polarization. (E) Cell population mapping (CPM) of up‐regulated genes in each group. (F) CPM of Inflammation‐associated cytokines and apoptosis genes in each group. (G–I) The correlation between mALFF values and the microglia proportion in AKI groups at different time courses. (J) The noninvasive ALFF index is negatively correlated with microglial proportion in AKI groups. The colors represent the AKI clusters at different time courses. *p < 0.05, **p < 0.01, ***p < 0.001.

Single‐cell suspensions from hippocampus brain tissues of each group were used to differentiate the populations of resident microglia by labeling with CD45 and CD11b. The results showed that cells in the AKI group had significantly higher expression of both CD45 and CD11b at 24 h compared to the control group and the M1 polarization (CD86^+^) significantly occurred at 48 h and peaked at 72 h (Figure 5A). Additionally, Figure 5B indicated that the percentage of activated microglia in the AKI group increased with the progression of AKI, ranging from 18.19 to 41.7% at 24 h, from 15.57 to 61.37% at 48 h, and from 16.85 to 82.57% at 72 h. Figure 5C showed that the percentage of M1 polarization significantly increased at 48 h and peaked at 72 h.

### 3.5 AKI induced microglial gene differential expression in Hippocampal

Processing of raw fastq files was carried out using zUMIs (version 2.4.1 or newer) and STAR (v2.5.4b) to generate expression profiles for the 3′ ends containing UMIs data. The Find_pattern: AAGCAGTGGTATCAACGCAGA GTACATTACT was specified for file1, and base_definition: UMI (32–39 bp) in the R1 file and cDNA (1–150 bp) in R2 file. UMIs were collapsed using a Hamming distance of 1. Rat cells were mapped against Rattus_norvegicus.mRatBN7.2 with CAST SNPs masked with N to avoid mapping bias. Quantification of data was done with gene annotations from Ensembl Rnor_6.0. Differential expression analysis was conducted using functions from the Bioconductor packages edgeR^38^ (v 3.36.0) and voom limma (v 3.36.0).^39^ Genes with the expression of at least one CPM (counts per million)^40^ were included in the analysis, and counts were normalized using the TMM method. Generalized linear models were used for differential expression analysis, using the functions glmQLFit and glmTreat31 with lfc = log2(CPM). Genes with absolute value of fold change greater than 2 were considered as differentially expressed.

Enriched GO terms when comparing the control and the AKI group included apoptosis and immune‐related signaling pathways (Figure 5D). Smart‐seq2 method identified 10,695 unique differentially expressed genes through hierarchical clustering, revealing a highly plastic transcriptome with 2892 up‐regulated genes and 2755 down‐regulated in the AKI group compared to the control (Figure 5E). Activated microglia produce proinflammatory cytokines, which are essential in pathological inflammatory processes (Figure 5F). The levels of proinflammatory cytokines such as IL‐1β and IL‐6 were validated in the hippocampus. Compared to the control group, different levels of IL‐1β and IL‐6 were found at 24 h and the differences were amplified from 48 to 72 h (Figure S2). This suggested that microglia were activated following AKI and released the proinflammatory cytokines accompanying with neuronal death, which suggested that microglia were involved in detrimental effects on neuronal function in AKI.

### 3.6 Correlation of mALFF with the microglia

Results from MRI and WM tests showed that AKI had a considerable effect on the brain, which is likely caused by alterations in microglia status. Pearson correlation was used to further analyze the relationship between ALFF index and the microglia. The results indicated a strongly negative correlation (*r* = −0.983) and with the activation of microglia, the correlation coefficient increased significantly (Figure 5G–I). These results suggest that more microglia activation is associated with lower mALFF in AKI groups, which could provide evidence for a time window of anti‐inflammatory therapy to protect neurons after AKI (Figure 5J).

### 3.7 AKI induced loss of Hippocampal Neurons

**FIGURE 6:**
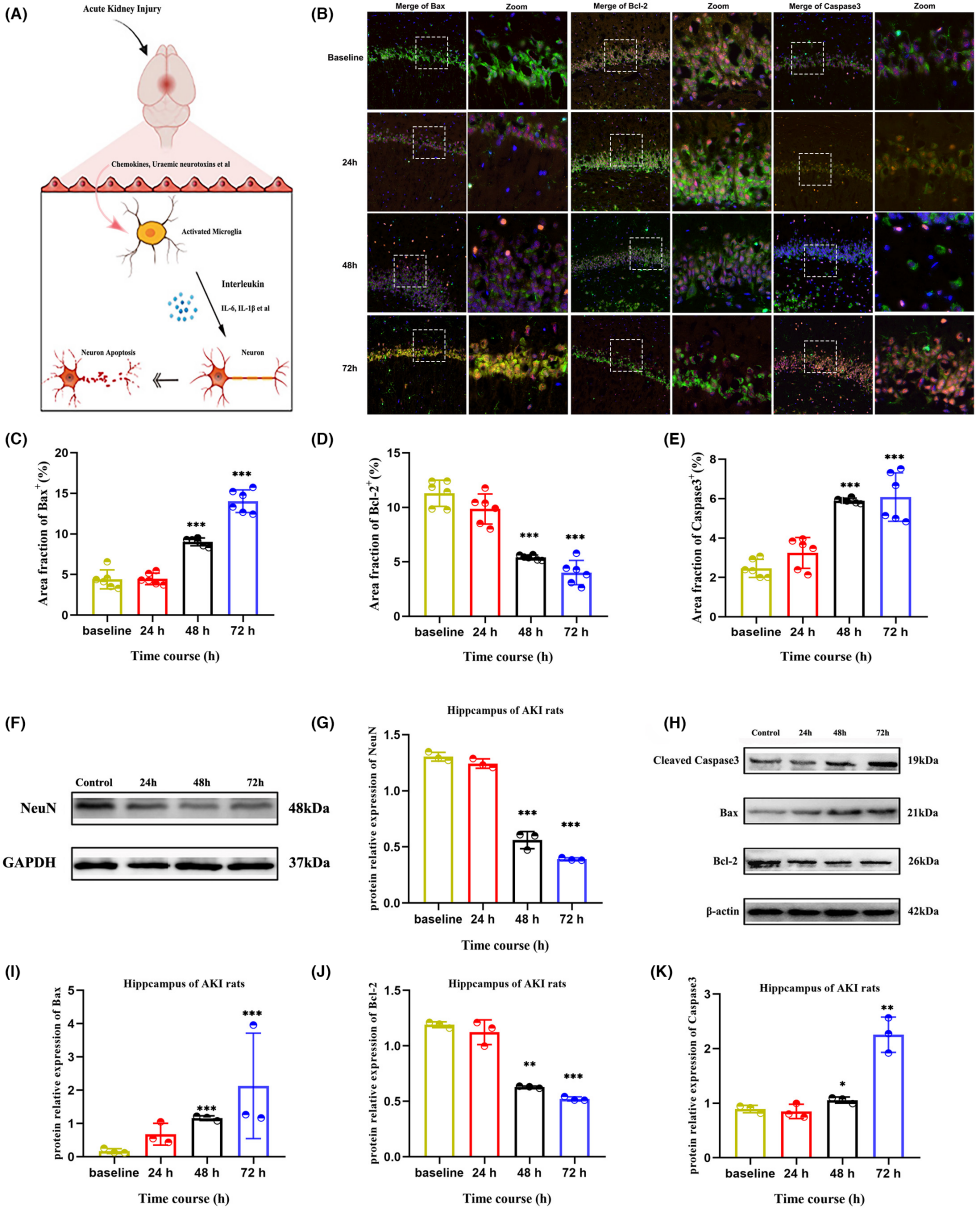
Effect of microglia on hippocampal neurons in AKI. (A) Schematic illustration of the microglia affecting the apoptotic neurons in AKI. (B) Confocal microscopy images of the density of microglia, neurons, and apoptosis relative protein at 24 h, 48 h, and 72 h after AKI. The white dashed boxes showed the zoomed area of the brain. The merge view was 20× and the zoom view was 60×. (C–F) Quantitative analysis of the area fractions of the Bax, Bcl‐2, and caspase3 signals calculated from the confocal microscopy images. (F, G) Represent the expression of neuron in each group. (H–K) Represent the apoptosis relative protein levels of cleaved caspase3, Bax, and Bcl‐2 in each group. Data were analyzed using ImageJ and were presented as the mean ± SD. *p < 0.05, **p < 0.01, ***p < 0.001.

Investigating the effect of microglia on the neurons in the AKI, we used immunohistochemistry and Western blot to measure the expression of NeuN (Figure 6A). Immunofluorescence signals and visualization of microglia, neuron, and apoptosis‐associated protein also indicated that AKI induced the aggregation of microglia and the incidence of neuronal apoptosis was significantly increased after 48 h post‐AKI (Figure 6B–E, Figure S1). Western blot showed a steady decrease in NeuN expression in AKI rats from 48 h to 72 h (Figure 6F). Quantitative analysis demonstrated that there was a 59.1% decrease in NeuN in the hippocampus of rats at 48 h and a 68.2% decrease at 72 h when compared with the baseline (Figure 6G). Western blot also revealed a significant increase in the levels of Bax and cleaved‐caspase3, and a decrease in Bcl‐2 compared to the control (Figure 6H–K, Figure S2). These results suggested that neuronal apoptosis occurred at 48 h and 72 h following AKI in the hippocampus.

## 4 DISCUSSION

In this study, we utilized resting‐state fMRI to investigate the effects of AKI on the central nervous system. The results indicated that dysfunction in the hippocampus may be a contributing factor to the cognitive impairments associated with AKI. Additionally, a mechanistic analysis revealed that an increased proportion of microglial activation may lead to neuronal loss through neuron apoptosis. Furthermore, a significant negative correlation was observed between microglial proportion and decreased average ALFF values in the aberrant brain clusters. This is the first study to assess brain functional alterations based on fMRI and to preliminarily detect the role of microglia on neurobiological mechanisms in AKI. The noninvasive ALFF analysis has the potential to quantitatively measure microglia, which could be beneficial for early prevention and targeted anti‐inflammatory therapy to protect neurons in clinical settings.

The application of amplitude of low‐frequency fluctuations (ALFF) analysis to the whole brain has been used to detect early functional deficits in central nervous system (CNS) diseases.^41^, ^42^ A study conducted on acute kidney injury (AKI) found that functional activity was significantly diminished in the hippocampus, a brain region particularly vulnerable to pathological conditions.^43^ Temporal analysis of AKI rat models further revealed that ALFF values of the hippocampus decreased at the onset of 48 h after AKI, which was in agreement with the results of the behavioral test.^44^, ^45^ Our further assessment revealed that the modified intensity of low frequency in the hippocampus could be attributed to impaired neuron function, which is consistent with cognitive behaviors. As discussed, evidence showed that the hippocampus is the first to detect disruption in AKI, and as the illness progresses, it may signal the beginning of AKI dementia. This implies that the potential advantages of rs‐fMRI may be used to detect not only neurodegeneration, but also acute neurotoxic brain inflammation.

Our research revealed that the neurological mechanism of the brain‐AKI connection was due to the release of inflammatory factors through the aggregation of microglia and the activation of the apoptosis pathway. As the microglia perform a constant surveillance of the brain environment and are activated in response to various signals, the proinflammatory microglia state results in the secretion of detrimental agents that trigger neuroinflammation and impedes the process of brain restoration.^46^ These proinflammatory factors infiltrate and induce the neuron loss, resulting in the impairment of learning and memory functions.^47^ Recent studies have demonstrated that microglial activation is a hallmark of neuropathology and can exacerbate brain injury in a neuropathic environment.^48^, ^49^, ^50^ We quantitatively analyzed the time course of microglia in the hippocampus using flow cytometry and RNA‐seq methods. The sequencing analysis suggested that microglia induced neuron impairment by stimulating the apoptosis signaling pathway and releasing inflammatory cytokines. To explore the cause of hippocampal neuron damage, we analyzed the levels of apoptosis‐related proteins and found that AKI caused neuron apoptosis at 48 h, which was consistent with the fMRI findings. Additionally, the upstream proinflammatory cytokines produced by activated microglia also led to reduced performance in the cognitive test in AKI rat models. We hypothesized that the cognitive impairments seen in AKI may be caused by activated microglia, leading to neuronal loss. The potential clinical application of fMRI may provide further insight into the mechanism of AKI‐related brain injury.

This study was the first to demonstrate the potential of rs‐fMRI to quantify microglia, which could be used to monitor neurological progression and serve as a biomarker for early neuroinflammation‐targeted treatment. Through correlation analysis, a significant negative correlation was found between ALFF values and the percentage of microglia, indicating the gradient changes in neuroinflammation during AKI progression. This suggests that fMRI can potentially provide a noninvasive method for assessing anti‐inflammatory therapy, which could protect neurons in a clinical setting.

Nevertheless, several limitations need to be mentioned in our study. Firstly, the mechanism of microglia inflammasome‐mediated neuron death is yet to be explored in detail, and further research is needed to identify potential therapeutic targets. Additionally, it is possible that other cellular changes in the brain following AKI might have a similar effect on rs‐fMRI, and thus, further investigation using multimodal fMRI technology is necessary to replicate and validate our findings.

## 5 CONCLUSION

This research indicated that AKI‐induced brain injury is a result of neuronal apoptosis due to microglia aggregation and inflammatory factor upregulation. Furthermore, the abnormal activity in fMRI can be utilized as a biomarker to measure the microglial proportion for neuroinflammation treatment and to assess cognitive impairment after AKI. As the connection between the brain and kidney is increasingly recognized, we emphasize that rs‐fMRI can assist in understanding the neural mechanisms of cognitive impairment, monitoring brain events, and improving clinical treatments following AKI.

## AUTHOR CONTRIBUTION

Ke Ren, Ziyang Yu, Huize Pang, Yifan Yang, Doudou Luo, Haiping Zheng, Zicheng Huang, and Mingxia Zhang performed all the experiments. Ziyang Yu, Huize Pang, and Ke Ren performed data analysis and wrote the manuscript. All authors read and approved the final manuscript.

## FUNDING INFORMATION

This study was supported by research grant from the National Natural Science Foundation of China (grant nos. 82071886 and 81703742).

## CONFLICT OF INTEREST STATEMENT

The authors of this manuscript declare no relationships with any companies, whose products or services may be related to the subject matter of the article. The manuscript has not been published in any other journal.

## GUARANTOR

The scientific guarantor of this publication is Dr. Ke Ren. The authors confirm that all methods were carried out in accordance with relevant guidelines and regulations.

## Supporting information


Table S1

Figure S1

Figure S2

## Data Availability

The data that support the findings of this study are available from the corresponding author upon reasonable request.
