# Supplementary material for: Microglia dysfunction drives disrupted hippocampal amplitude of low frequency after acute kidney injury
Source: CNS Neurosci Ther. 2023 Jul 19;30(2):e14363. doi: 10.1111/cns.14363 (PMC10848109; doi:10.1111/cns.14363)
Supplement: Supplementary file 1 — Table S1 [file CNS-30-e14363-s003.docx]

E1. Antibody information

| **Protein name** | **Product code** | **Manufacturer** |
| --- | --- | --- |
| NeuN | ab177487 | Abacm |
| Cleaved Caspase3 | ab231289 | Abacm |
| Bax | ab32503 | Abacm |
| Bcl-2 | ab32124 | Abacm |
| β-actin | ab6276 | Abacm |
| GADPH | ab8245 | Abacm |
| KIM-1 | A2831 | ABclonal |
| Secondary antibody | ab288151 | Abcam |
| Interleukin-1β | ab100712 | ABclonal |
| Interleukin-6 | ab100712 | ABclonal |
