# Supplementary figures and images for: Microglia dysfunction drives disrupted hippocampal amplitude of low frequency after acute kidney injury
Source: CNS Neurosci Ther. 2023 Jul 19;30(2):e14363. doi: 10.1111/cns.14363 (PMC10848109; doi:10.1111/cns.14363)

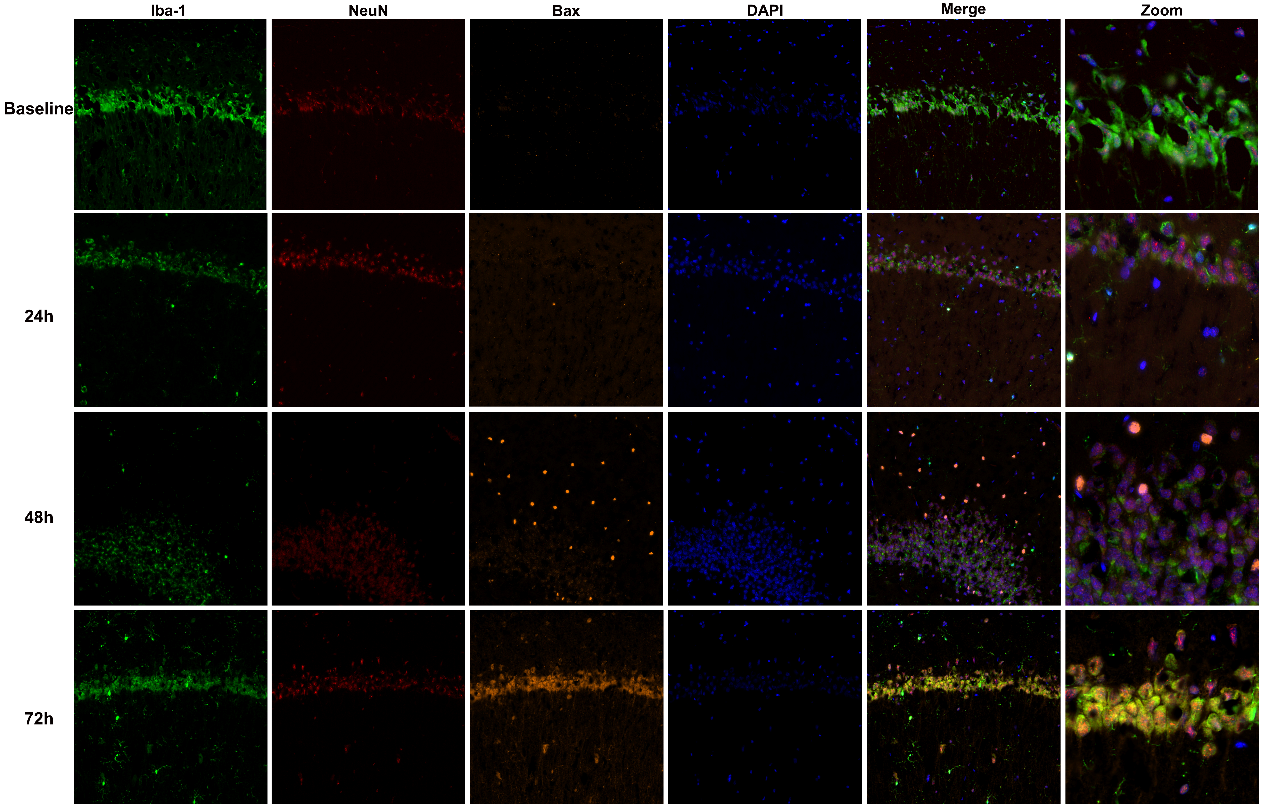


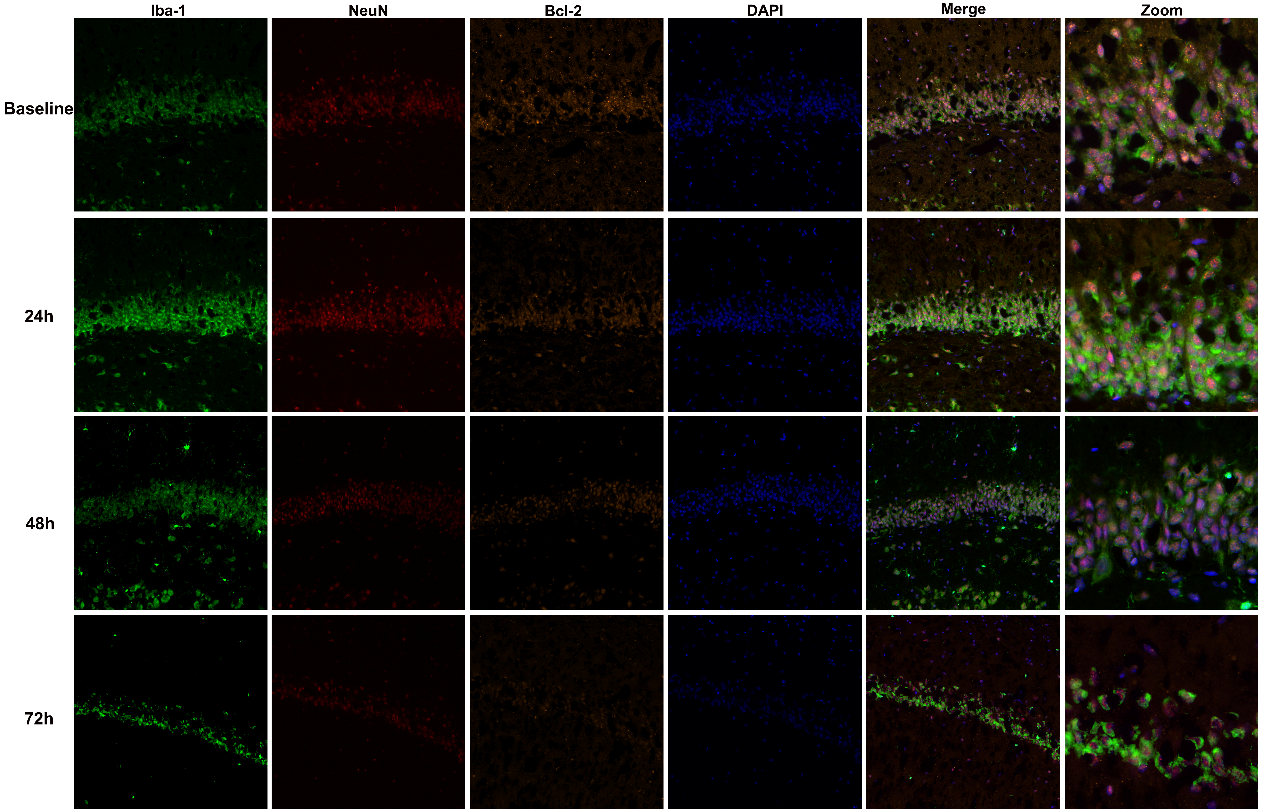


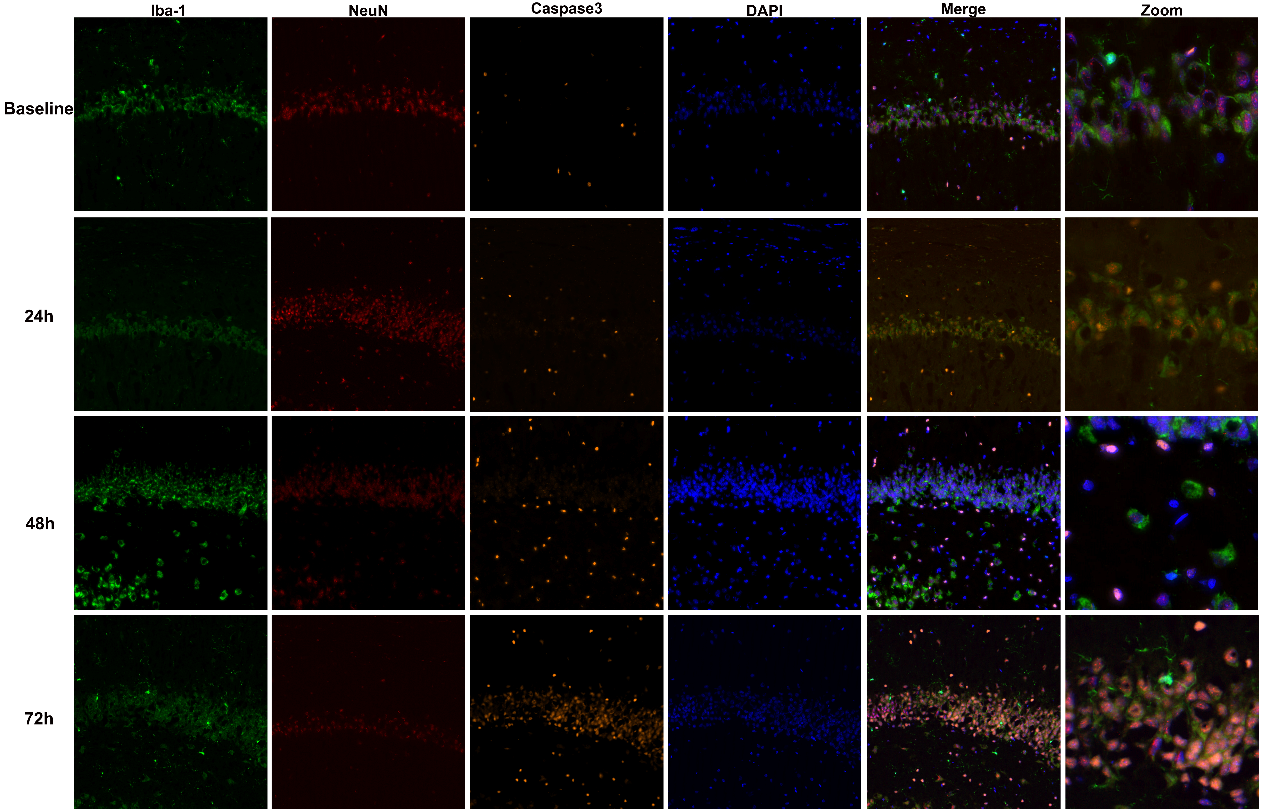


**Supplementary Figure. S3 | Complete Confocal microscopy images.**

Source data for Figure 6b.

Supplement: Supplementary file 2 — Figure S1 [file CNS-30-e14363-s001.docx]
