# Supplementary material for: Microglia dysfunction drives disrupted hippocampal amplitude of low frequency after acute kidney injury
Source: CNS Neurosci Ther. 2023 Jul 19;30(2):e14363. doi: 10.1111/cns.14363 (PMC10848109; doi:10.1111/cns.14363)
Supplement: Supplementary file 3 — Figure S2 [file CNS-30-e14363-s002.docx]

**(a) NeuN: 48kDa**


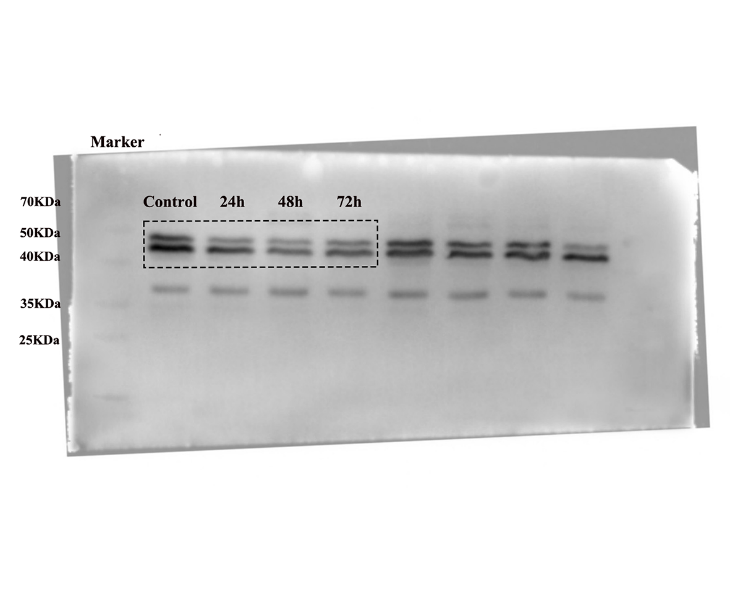


**(b) GAPDH: 37kDa**


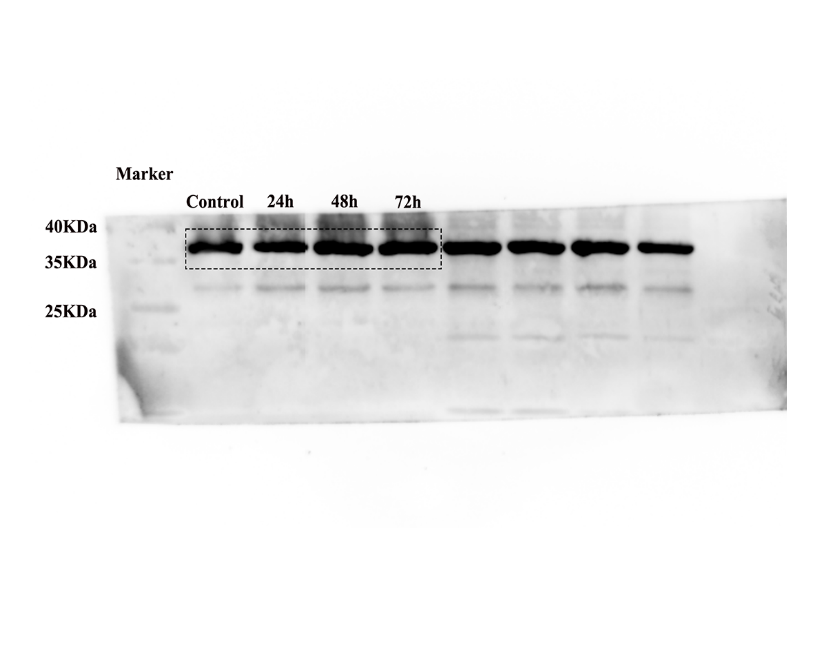


**(c) Cleaved Caspase3: 19kDa**


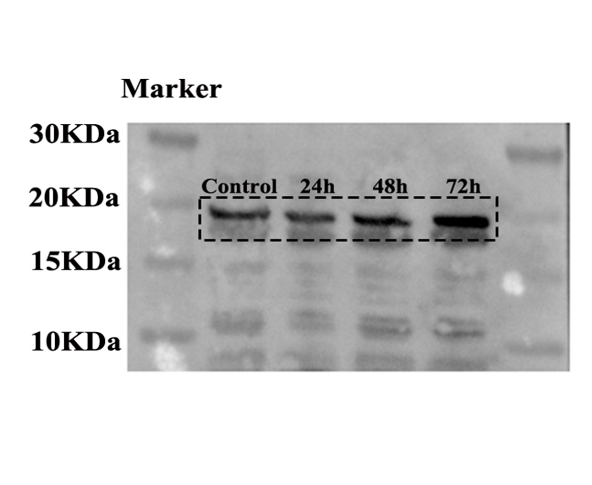


**(d) Bax: 21kDa**


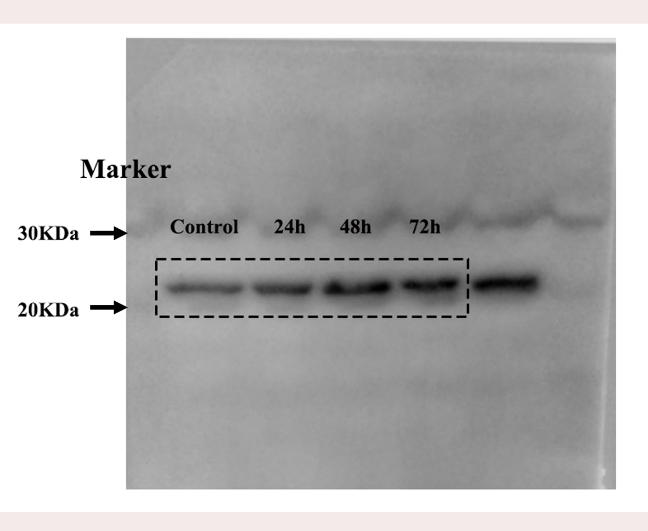


**(e) Bcl-2: 26kDa**


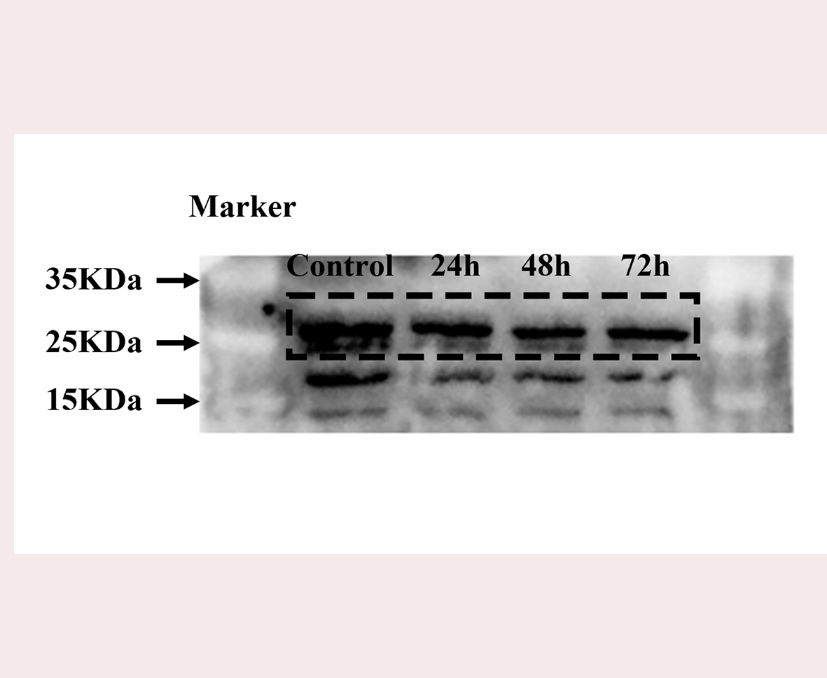


**(f) β-actin: 42kDa**


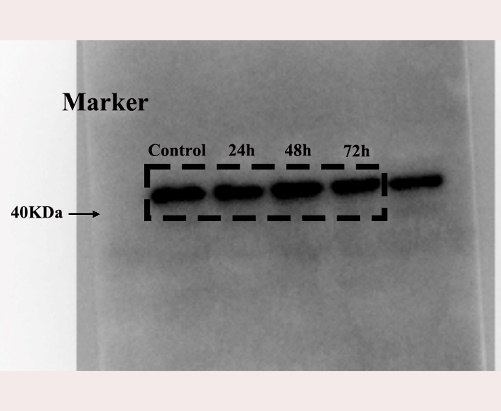


**Figure Legends | Complete uncropped gel and western blot images of figures.**

(a, b) Source data for Figure 6a.

(c-f) Source data for Figure 6c.
